# Solitary Fibrous Tumour of the Pleura Presenting as a Spontaneous Massive Haemothorax

**DOI:** 10.1155/2014/139404

**Published:** 2014-01-09

**Authors:** Giampiero Negri, Alessandro Bandiera, Paola Ciriaco, Giulio Melloni, Angelo Carretta, George Ian Cremona, Piero Zannini

**Affiliations:** ^1^Department of Thoracic Surgery, San Raffaele Scientific Institute, Via Olgettina 60, 20132 Milan, Italy; ^2^Department of Pulmonary Diseases, San Raffaele Scientific Institute, Milan, Italy

## Abstract

Solitary fibrous tumours of the pleura are rare neoplasms. These tumours are generally asymptomatic and incidentally diagnosed. Symptoms, if present, are nonspecific such as cough, dyspnea, and chest pain. This report describes the case of a 38-year-old woman admitted to our department after the onset of a right massive spontaneous haemothorax requiring emergency surgical treatment. Intraoperatively a bleeding pleural mass was found to be the cause of the haemothorax. The tumour was successfully resected and the patient made an uneventful recovery. Histological examination revealed the mass to be a solitary fibrous tumour of the pleura.

## 1. Introduction

Solitary fibrous tumours of the pleura (SFTP) are rare tumours of mesenchymal origin. SFTP may arise from the visceral or the parietal pleura and may present as small nodules or large masses. For the most part, SFTP are chance findings as they are usually asymptomatic. Symptoms, when present, are generally due to a mass effect and compression and are the same as those observed with other intrathoracic masses, namely, cough, thoracic pain, and dyspnea. Other symptoms, such as haemoptysis, and paraneoplastic syndromes and most commonly a refractory hypoglycemia have been reported [1–4]. In a few cases serous pleural effusion may also be associated with SFTP. To the best of our knowledge, SFTP presenting as a spontaneous haemothorax has been very rarely described in the literature [5–7]. This report describes the case of a 38-year-old woman admitted to our Department of Thoracic Surgery for a right massive spontaneous haemothorax. Emergency surgical thoracotomy revealed a bleeding mass arising from the parietal pleura to be the cause of the haemorrhage. Resection of the mass halted the bleeding and subsequent pathological evaluation revealed it to be a benign SFTP.

## 2. Case Report

**Figure 1:**
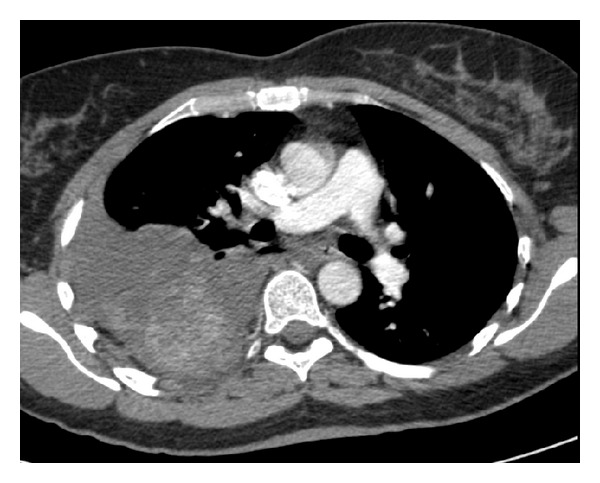
CT scan showing a right pleural effusion consistent with a haemothorax due to recent and active bleeding associated with a paravertebral hyperdense formation (7 cm in diameter) suspected to be a parietal neoplasm.

**Figure 2:**
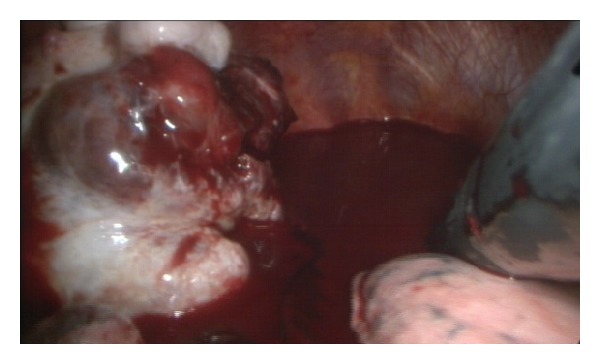
Intraoperative detection of a bleeding pleural tumour surrounded by a massive haemothorax.

A 38-year-old woman was referred to our Department of Thoracic Surgery for a right massive spontaneous haemothorax. She was initially assessed by the Emergency Department of another hospital where she complained of acute right chest pain and dyspnea. A chest radiograph performed on initial evaluation revealed a right pleural effusion. Computed tomography of the chest indicated the nature of the pleural effusion to be consistent with a haemothorax. A parietal paravertebral opacity, suspected to be an intrathoracic tumour surrounded by blood clots, was detected (Figure 1). The patient was urgently transferred to our department for surgical evaluation. The clinical status of the patient rapidly worsened with hypotension and tachycardia. Blood tests revealed an anaemia requiring blood transfusion. The patient was transferred urgently to the operating theatre where a preliminary right thoracoscopy was performed. After the removal of 1500 mL of blood and blood clots, a large paravertebral bleeding tumour was detected at the level of the fourth and fifth ribs (Figure 2). No adhesions were found between the tumour and the visceral pleura. A right sixth intercostal space posterolateral thoracotomy was then performed to expose and manage the tumour which was bluntly dissected away from the endothoracic fascia through an extrapleural plane and resected, thus arresting the bleeding. The patient had an uneventful recovery in the postoperative period and remained asymptomatic till the last followup, six months after surgery.

Pathological examination revealed a SFTP with fibrous areas and low cellularity. None of the previously described histological criteria for malignancy [1] (high cellularity, presence of necrosis, mitotic count of more than 4 mitosis/10 high-power fields, and presence of nuclear atypia) were observed so the final response was a benign SFTP.

## 3. Discussion

Spontaneous haemothorax is an unusual clinical condition. The most common cause is pneumothorax as bleeding can result from the rupture of vascularized adhesions between the visceral and parietal pleura or from the rupture of vascularized bullae. Less frequently the causes of spontaneous haemothorax may include coagulopathies, vascular ruptures, endometriosis, and neoplasias [8]. In patients, affected by SFTP, anecdotal cases of haemothorax have been described [5–7]. However, massive bleeding, requiring emergency surgical treatment, is to be considered an extremely rare event. 

Computed tomographic scan is a highly accurate diagnostic study for pleural blood effusion and our patient was able to undergo the exam thanks to her initial clinical stability at the referring hospital. The presence of an intrathoracic tumour was suspected. 

In most cases of haemothorax, the insertion of a large calibre chest tube drainage is usually the initial step of the treatment in stable patients. In our patient, an immediate surgical approach was performed taking into account the suspected diagnosis of an intrathoracic bleeding tumour at CT scan and the unstable haemodynamic clinical status at the time of our evaluation.

In the literature [9], massive haemothorax in haemodynamically unstable patients has been reported to be a contraindication for video assisted thoracoscopic surgery. Our patient was able to tolerate single lung ventilation and a preliminary exploration by thoracoscopy was performed only to confirm the presence of a tumour and to decide the level of the thoracotomy. By means of the subsequent open access the tumour was found not to infiltrate the chest wall and was rapidly resected. On removal of the tumour, the bleeding was immediately arrested. Taking into account the absence of adhesions between the tumour and the visceral pleura, this finding confirms that the cause of the bleeding was the tumour itself. Although the association between intrathoracic tumours and bloody pleural effusion tends to suggest histological malignancy, in our patient the pathological analysis demonstrated a benign SFTP.

This report confirms that a benign SFTP may present with uncommon clinical features and that a massive spontaneous haemothorax may be the first presentation of this type of neoplasm. Moreover, even a benign tumour may present with a massive haemothorax and may be lethal if not promptly managed.
